# Failure modes of robotic-assisted arthroplasty platforms in Australia: a descriptive analysis of TGA adverse event reports

**DOI:** 10.1007/s11701-026-03890-9

**Published:** 2026-09-03

**Authors:** Dylan Rowe, Mariam Rowe

**Affiliations:** 1 School of Medicine and Dentistry, Griffith University, Gold Coast, QLD Australia; 2 Princess Alexandra Hospital, QLD Brisbane, Australia; 3 Faculty of Medicine, University of Queensland, Herston, QLD Brisbane, Australia

**Keywords:** Robotic arthroplasty, Adverse events, MAKO, ROSA, NAVIO, Cori

## Abstract

Robotic-assisted arthroplasty is increasingly performed using technically distinct platforms. The types of device problems reported for different robotic arthroplasty platforms have not been characterised using Australian national surveillance data. The TGA Database of Adverse Event Notifications (DAEN) was searched for robotic arthroplasty adverse events reported between July 2012 and December 2025. Failure modes were extracted from the DAEN classification, and qualitative review of event narratives was performed independently by two authors. One hundred and forty reports were identified across five platforms: ROSA Recon (*n* = 46, 32.9%), MAKO (*n* = 45, 32.1%), NAVIO/Cori (*n* = 33, 23.6%), Zimmer CAS/ORTHOsoft (*n* = 15, 10.7%) and VELYS (*n* = 1, 0.7%). The retrieved reports for each platform contained different mixtures of DAEN failure-mode categories. MAKO reports commonly described registration-related inaccuracies and resection beyond planned boundaries despite haptic guidance, whereas ROSA Recon reports predominantly described discrepancies between planned and achieved bone resection. Zimmer CAS/ORTHOsoft reports were mostly navigation-related inaccuracies, while NAVIO/Cori demonstrated frequent software-related failures, including an 11-report cluster describing failure of the femoral notch-warning algorithm. Australian adverse event reports contained platform-specific patterns of reported device problems. Qualitative review identified recurring technical and software-related signals that were not always apparent from DAEN failure-mode coding alone. Awareness of these platform-specific failure modes may assist surgeons in recognising device-related problems during surgery and should inform training for surgeons transitioning between robotic platforms.

## Introduction

Robotic-assisted arthroplasty is increasingly performed throughout Australia, with major surgical centres now routinely using multiple platforms for total knee, total hip and unicompartmental arthroplasty [1, 2]. Contemporary systems include the MAKO robotic arm (Stryker), ROSA Recon Platform (Zimmer Biomet), NAVIO/Cori (Smith & Nephew) and VELYS (DePuy Synthes). Robotic-assisted arthroplasty has demonstrated improved accuracy of implant positioning compared with conventional instrumentation across multiple platforms, supporting its increasing clinical adoption [3–8]. As utilisation expands, understanding the circumstances in which these systems fail becomes increasingly important for patient safety, surgeon training and system design.

These platforms employ fundamentally different approaches to imaging, registration, navigation and robotic assistance. MAKO uses CT-based preoperative planning with haptic boundary control, ROSA Recon combines optical tracking with a motorised robotic arm, NAVIO/Cori is a handheld image-free system with software-defined safety constraints, and VELYS is an imageless optically tracked robotic-assisted platform. These architectural differences likely influence how each system fails during surgery and, consequently, the failure modes that surgeons and trainees should recognise, particularly in centres where multiple robotic platforms are used.

Current research of adverse event surveillance in robotic arthroplasty has relied predominantly on the United States Food and Drug Administration’s Manufacturer and User Facility Device Experience (MAUDE) database. Most published analyses have examined individual platforms, identifying hardware failures, inaccurate bone resection and robotic arm malfunction among the most reported adverse events [9–11]. One study by Zheng et al. examined robotic knee arthroplasty platform adverse events using MAUDE, finding an overall increase in adverse events over the past decade, and reporting inappropriate bone resection as the most frequent surgical complication [12]. Broader analyses of robotic surgery adverse events have been dominated by the da Vinci platform and are therefore of limited relevance to orthopaedic robotic systems [13].

In Australia, the Therapeutic Goods Administration (TGA) Database of Adverse Event Notifications (DAEN) has publicly reported medical device adverse events since July 2012 [14]. Unlike MAUDE, the DAEN is an Australian surveillance database that is almost entirely industry-sourced and primarily captures suspected device malfunctions rather than clinical outcomes. However, this reporting landscape is likely to evolve following the introduction of mandatory adverse event reporting by Australian healthcare facilities in March 2026 [15]. To our knowledge, the Australian DAEN has not previously been analysed for robotic arthroplasty devices.

The aims of this study were to characterise the types of device problems and failure modes reported to the Australian TGA DAEN for robotic-assisted arthroplasty platforms, and to identify recurring or clinically relevant signals within the reported event narratives. Our secondary aim was to describe platform-specific examples of reported device problems that may be relevant to surgical education, post-market surveillance and future investigation.

## Methods

### Data source

The Therapeutic Goods Administration (TGA) Database of Adverse Event Notifications (DAEN) was searched on 1 June 2026 for reports lodged between 1 July 2012 and 31 December 2025. Searches used combinations of keywords (robot*, MAKO, ROSA, NAVIO, CORI, VELYS, Zimmer, Stryker and Smith & Nephew). Reports were included where the GMDN term, trade name, manufacturer or event description was consistent with an orthopaedic arthroplasty application. Reports relating exclusively to spinal, neurosurgical or non-orthopaedic robotic systems were excluded.

All retrieved reports were reviewed independently by both authors (D.R. and M.R.) for study eligibility, with disagreements resolved by consensus. No disagreements occurred during analysis. Reports were reviewed for potential duplicate notifications using report date, device, manufacturer and event description, with all duplicates removed prior to analysis. Ethics approval was not required because all data were publicly available and de-identified.

National musculoskeletal robotic procedure volumes were obtained from the Australian Institute of Health and Welfare National Hospital Morbidity Database Procedures Data Cubes (ACHI 96233-01) for contextual reference [16].

### Classification

Reports were grouped based on platforms according to the associated GMDN, device name, and manufacturer (MAKO, ROSA, NAVIO/CORI, VELYS, CAS/ORTHOsoft). NAVIO/Cori was grouped as the robotic platform, associated accessories and CoriGraph preoperative planning software. Failure mode was extracted directly from the DAEN ‘Event Type’ field of each report. Platform-specific findings were evaluated using the ‘Event Description’ field of each report, classification data were reviewed independently by both authors and disagreements were resolved by consensus.

### Statistical analysis

Descriptive statistics (counts and proportions, stratified by platform and failure mode) were calculated in Microsoft Excel (Microsoft Corporation, Redmond, WA, USA). No inferential comparisons between platforms were performed, given the passive surveillance design of the DAEN and the absence of reliable platform-level utilisation data needed to calculate a denominator. Consistent with the TGA’s own stated limitations for this database, all percentages reported in this study represent the proportion of reports within a given platform or category (i.e., report composition) and cannot be interpreted as the incidence or likelihood of an adverse event occurring among device users [14]. Report submission dates were tabulated to identify temporal clustering.

## Results

### Overview

**Fig. 1:**
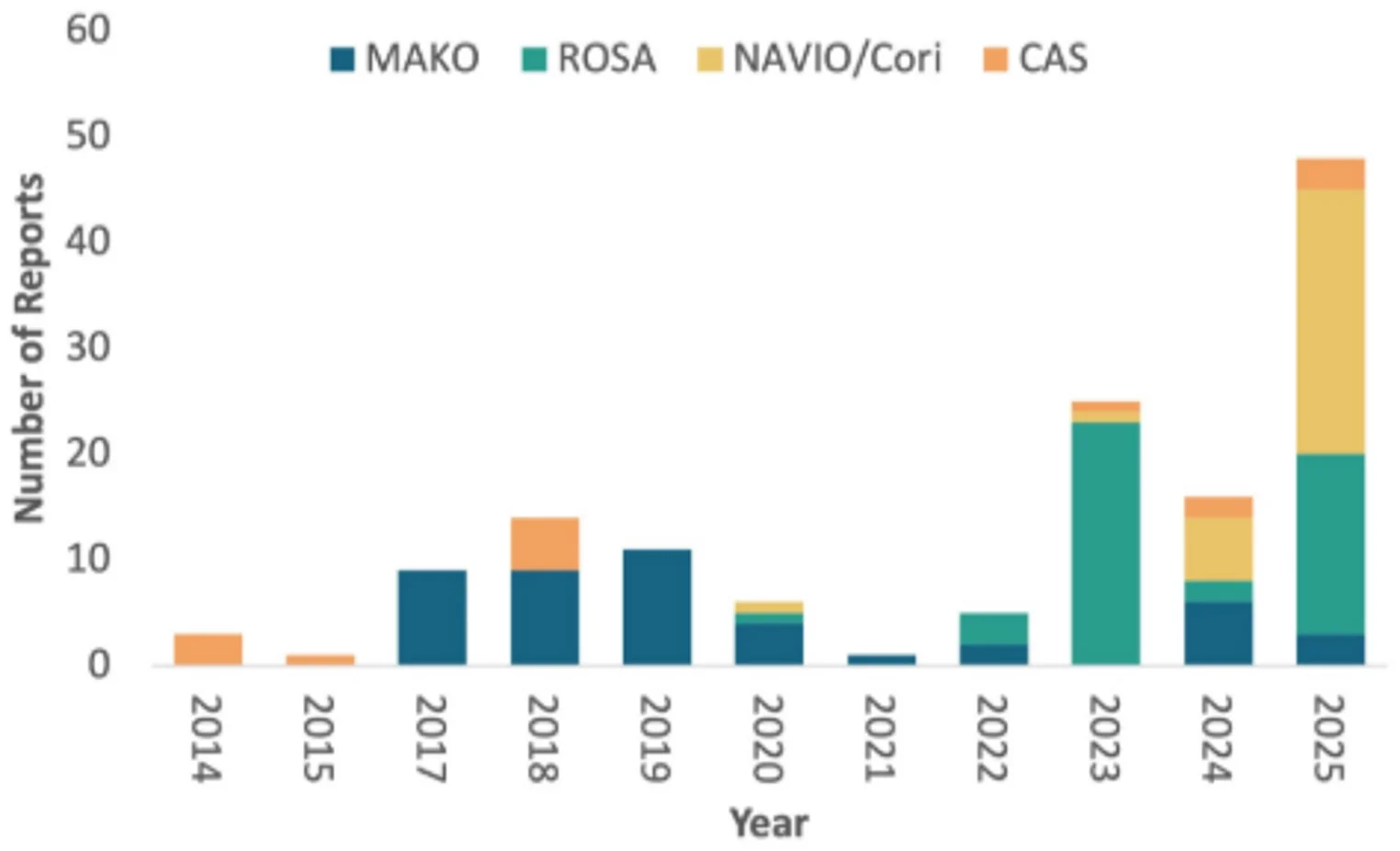
Annual number of adverse event reports concerning robotic arthroplasty platforms submitted to the TGA DAEN, 2012–2025 (*n* = 140). No reports identified for 2012, 2013, or 2016. VELYS (*n* = 1) excluded for visualisation

A total of 140 adverse event reports met the inclusion criteria. Industry submitted 137 reports (97.9%), with 3 (2.1%) reported by Healthcare Services. Annual reports were low between 2012 and 2022 (0–14 reports/year), before increasing to 26 in 2023, 16 in 2024 and 48 in 2025 (Fig. 1). No reports were identified for 2012, 2013, or 2016. National musculoskeletal robotic procedure volumes increased from 20,876 in FY2022–23 to 28,008 in FY2023–24 (AIHW ACHI 96233-01). However, denominator data were only available for these two financial years, and therefore could not be used to calculate longitudinal event rates across the study period. Earlier AIHW reporting grouped robotic procedures across multiple specialties, preventing isolation of arthroplasty-specific volumes [16]. When grouped by platform, the reports comprised ROSA Recon (*n* = 46, 32.9%), MAKO (*n* = 45, 32.1%), NAVIO/Cori (*n* = 33, 23.6%), Zimmer CAS/ORTHOsoft (*n* = 15, 10.7%) and VELYS (*n* = 1, 0.7%) (Table 1).

**Table 1:** Adverse event reports for robotic arthroplasty systems in the TGA DAEN, 2012–2025 (*n* = 140)

Platform (Manufacturer)	ARTG Listed Date	*N* = 140	%
ROSA Recon Platform (Zimmer Biomet)	Jun 2019	46	32.9
MAKO Robotic Arm (Stryker)	May 2014	45	32.1
NAVIO/Cori (Smith & Nephew)	Aug 2017	33	23.6
Zimmer CAS / ORTHOsoft (Zimmer Biomet)	Nov 2005	15	10.7
VELYS (DePuy Synthes)	Jun 2021	1	0.7

*ARTG* Australian Register of Regulatory Goods

### Failure modes

Among the four platforms with sufficient report numbers, the composition of failure-mode categories within each platform’s own reports differed (Table 2). VELYS has been removed from analysis due to only having one report available. Usability and human factors events predominated for MAKO (19/45, 42%) and Zimmer CAS/ORTHOsoft (8/15, 53%), whereas output problems were the most common failure mode for ROSA (9/46, 20%) and NAVIO/Cori (9/33, 27%). NAVIO/Cori accounted for five of the six protective measures failures identified across the dataset (83%).

**Table 2:** Characteristics of adverse event reports within each robotic platform

Failure Mode Category (DAEN)	MAKO (*n* = 45) *n* (%)	ROSA (*n* = 46) *n* (%)	CAS (*n* = 15) *n* (%)	NAVIO/Cori (*n* = 33) *n* (%)
Usability / Human Factors	19 (42)	7 (15)	8 (53)	1 (3)
Output Problem	0 (0)	9 (20)	0 (0)	9 (27)
Protective Measures Problem	1 (2)	0 (0)	0 (0)	5 (15)
Mechanical Failure	5 (11)	6 (13)	1 (7)	2 (6)
Material / Component Failure	1 (2)	3 (7)	3 (20)	4 (12)
Activation / Positioning / Separation	2 (4)	4 (9)	1 (7)	2 (6)
Calibration Failure	3 (7)	3 (7)	0 (0)	4 (12)
Software / Computer Failure	1 (2)	1 (2)	1 (7)	4 (12)
Other / Unclassified	13 (29)	13 (28)	1 (7)	2 (6)

VELYS (*n* = 1) excluded from comparative analysis. Percentages represent the proportion of each platform’s own reports assigned to a given category. ‘Other/Unclassified’ includes unclassified events, patient-device interaction, connection problems, temperature-related issues, and electrical/electronic failures

### Platform-specific event descriptions

Platform-specific event patterns identified through narrative review of report event descriptions are summarised in Table 3. Two findings stand out. ROSA Recon had a larger cluster of resection-accuracy events (27/46, 59%) than the “Output Problem” code alone captures (9/46, 20%), spread across eight different failure mode categories. For Zimmer CAS/ORTHOsoft, 8 of 15 reports (53%) described a discrepancy between the navigation system’s displayed result and the actual surgical outcome. Most often this was lost hip offset or malalignment, with no accompanying intraoperative error message in any of the eight.

**Table 3:** Platform-specific adverse event report patterns

Platform	Pattern	*n* (%)	Event Description Example
**MAKO (n = 45)**	Registration/setup error	6 (13)	Preoperative imaging allocated to contralateral side; registration discrepancy identified before cutting
	Resection beyond planned limits	9 (20)	Cuts 1.5–3 mm deeper than intended despite haptic boundary activation; system restart/re-homing required intraoperatively
	Arm lock during resection	2 (4)	Arm locked within joint, recurring after reboot, requiring re-homing and repeat cut pass
	Burr/handpiece malfunction	2 (4)	Burr detached from motor and continued spinning, causing unintended femoral defect
	Acetabular fracture	3 (7)	Periprosthetic fracture during component impaction
**ROSA Recon (n = 46)**	Resection accuracy discrepancy	27 (59)	9.5 mm planned resection measured 13 mm on physical inspection despite intraoperative validation
	Hardware failure	6 (13)	Camera support chain fractured; camera dropped intraoperatively
**CAS (n = 15)**	Silent alignment/offset discrepancy	8 (53)	Navigation indicated offset restored; postoperative imaging and clinical findings showed otherwise, with no error message
	Pin fracture, retained fragment	4 (27)	Navigation pin tip fractured, fragment retained in bone
	System/software failure	3 (20)	Checkpoint software failure causing procedural delay
**NAVIO/Cori (n = 33)**	Sizing/calibration inaccuracy	5 (15)	Implant overlay centred over medial condyle, substantially undersized
	Screen/output discrepancy	6 (18)	Device blanked out approximately six times before tibial cut
	Mechanical/ component failure	3 (9)	Tracker displacement during resection, converted to manual technique

Patterns shown account for 22/45 (49%) of MAKO reports, 33/46 (72%) of ROSA Recon reports, 15/15 (100%) of CAS reports, and 14/22 (64%) of NAVIO/Cori reports. Remaining reports did not fit a clearly defined pattern or category and are not itemised

### NAVIO/Cori notch-warning cluster

Between 23 January and 28 April 2025, 11 reports described failure of the NAVIO/Cori femoral notch-warning algorithm during total knee arthroplasty (Table 4). In each case, the software failed to alert the surgeon when the planned anterior femoral resection risked notching. Seven reports were submitted on a single day (13 February 2025), consistent with a batch notification by industry. Notably, although all 11 reports related to the same underlying software issue, they were distributed across four DAEN failure mode categories: Protective Measures Problem (*n* = 5), Software/Computer Failure (*n* = 3), Output Problem (*n* = 2) and Mechanical Failure (*n* = 1).

**Table 4:** NAVIO/Cori femoral notch-warning algorithm–related adverse event reports in the TGA DAEN, January–April 2025 (*n* = 11)

Report No.	Date	Event Description
104,713	23 Jan 2025	Notch warning did not appear during planning
108,589	24 Jan 2025	Warning Error
104,903	13 Feb 2025	Warning did not appear when it should have
104,910	13 Feb 2025	Notch warning absent; tibia struggling to register
104,948	13 Feb 2025	Notch warning did not appear; minor delay to surgery
104,958	13 Feb 2025	Notch warning did not appear
104,962	13 Feb 2025	Notch warning not displayed
104,966	13 Feb 2025	Software did not show notch warning during planning
105,410	13 Feb 2025	Notch warning absent; aggressive anterior cut made and manually smoothed
108,257	28 Apr 2025	Anterior cut would notch
108,315	28 Apr 2025	Notch warning did not appear

## Discussion

The principal finding of this study is that within the reports received by the TGA, robotic arthroplasty platforms do not share a common failure profile. Each platform’s reports showed a characteristic composition of adverse event types that was broadly consistent with its underlying design. These differences have important clinical implications, as surgeons increasingly transition between robotic platforms during training and practice. Prior experience in robotic-assisted surgery has been shown to shorten the learning curve associated with adopting an alternative robotic platform [17, 18]. However, our findings suggest that when transitioning between robotic systems, surgeons should anticipate device-related issues that are unique to the architecture of each platform.

The DAEN failure mode categories were informative but did not always capture the underlying nature of an adverse event. The NAVIO/Cori femoral notch-warning cluster comprised 11 reports describing the same software-related issue, yet these were distributed across four different DAEN failure mode categories. Similarly, for ROSA Recon, only 9 reports (20%) were coded as Output Problem, whereas qualitative review identified 27 reports (59%) describing discrepancies between the planned and achieved resection across eight different coding categories. These findings suggest that qualitative review of event descriptions provides important context beyond the assigned failure mode classification.

The observed differences between platforms were broadly consistent with their underlying operating principles. MAKO reports commonly involved registration-related inaccuracies and resections extending beyond the planned boundaries despite haptic guidance. ROSA Recon reports were dominated by discrepancies between planned and achieved bone resection, consistent with its dependence on continuous optical tracking. Although legacy Zimmer CAS uses the same optical tracking principle, it functions as a navigation system rather than a robotic arm, and most reports described discrepancies between the intraoperative navigation display and the eventual surgical outcome rather than equipment malfunction. Pin fracture with retained fragment was also a recurrent CAS finding (4/15, 27%), consistent with recognised risks of pin-site complications in navigated and robotic-assisted knee arthroplasty [19, 20]. In contrast, NAVIO/Cori relies heavily on software-defined planning and safety functions, reflected by its concentration of software-related reports and the femoral notch-warning cluster. These architectural differences are consistent with prior comparisons showing that component positioning and alignment outcomes vary between robotic platforms even when using comparable surgical philosophies [21].

The femoral notch-warning cluster identified in NAVIO/Cori warrants particular attention. Eleven reports described failure of the notch-warning algorithm to identify planned anterior cortical violation. Although only one report documented an intraoperative consequence, anterior femoral notching is a recognised risk factor for supracondylar periprosthetic fracture [22]. This finding demonstrates that software-generated safety alerts may themselves be susceptible to failure, with the potential to contribute to patient harm if the missing alarm is not recognised intraoperatively as identified in previous research [23, 24].

These findings may provide useful material for platform-specific training, particularly for familiarising surgeons and trainees with device-related problems that may be encountered during robotic-assisted procedures. Current training in Australia remains largely vendor-directed, with no nationally standardised orthopaedic curriculum for robotic systems [25, 26]. The present findings suggest that platform-specific failure modes should form part of surgical training. Familiarity with one robotic platform should not be assumed to translate to another, as the failure patterns identified for MAKO, ROSA Recon and NAVIO/Cori were fundamentally different.

Comparison with the international literature demonstrates both similarities and differences. The predominance of output problems and resection accuracy issues for ROSA Recon and NAVIO/Cori is consistent with Zheng et al., who identified inappropriate bone resection as the most common adverse event across robotic knee arthroplasty platforms [12]. MAKO arm-locking events parallel the unexpected robotic arm movement reported by Pagani et al. [10]. Conversely, the dominance of hardware failures reported by Graefe et al. for robotic total hip arthroplasty was not observed in the Australian dataset, while the increasing number of Australian reports contrasts with the declining incidence described by Costello et al. [9, 11]. This may potentially be explained by the Australian ecosystem being in a less mature phase of adoption. Additionally, differences in reporting systems, platform utilisation and adoption stage may contribute to these observations, and direct comparison between DAEN and MAUDE should be interpreted cautiously.

This study has several limitations. First, the relative proportions of reported event types should not be interpreted as representing their relative frequency in clinical practice. A higher proportion of a particular event type within reports for one platform may reflect differences in reporting behaviour, platform utilisation, sponsor reporting practices, device age, market penetration, clinical application, or the underlying likelihood of the event itself. Although this study included the complete Australian DAEN dataset for the study period, the number of reports for individual platforms was relatively small, limiting assessment of less common event types. In addition, the included robotic platforms were introduced into Australian practice at different time points and likely represent different stages of clinical adoption. Differences in surgeon familiarity, procedural volume, and market penetration may therefore have influenced reporting patterns independently of platform architecture. The DAEN is predominantly industry-sourced and consequently captures suspected device-related events rather than providing a complete record of clinical outcomes. The specific procedure being undertaken at the time of an event could not be determined for most reports because of limited information, and failure-mode coding may vary between sponsors. Finally, platform-specific sample sizes and the absence of reliable utilisation denominators precluded inferential statistical comparison between platforms.

Future studies should determine whether platform-specific awareness of failure modes can be incorporated into robotic arthroplasty training and whether this improves recognition and management of device-related problems during platform adoption. Improved linkage between adverse event reporting, procedural denominator data and the Australian Orthopaedic Association National Joint Replacement Registry may also allow estimation of platform-specific event rates and correlation with patient outcomes [27].

## Conclusion

Robotic arthroplasty platforms in Australia are associated with distinct patterns in the types of adverse events reported to the TGA DAEN, broadly consistent with differences in system architecture and workflow. Qualitative review of adverse event narratives identified platform-specific failure patterns that were not apparent from DAEN failure mode coding alone. As robotic arthroplasty becomes more widely adopted, surgeons and trainees should recognise that failure patterns differ between platforms and prior knowledge is not necessarily transferable. Awareness of these platform-specific failure modes may facilitate earlier recognition of device-related problems during surgery, inform platform-specific training, and provide a benchmark for future surveillance as robotic usage matures in Australia.

## Data Availability

No datasets were generated or analysed during the current study.
